# Supplementary material for: A novel modulator of IL-6R prevents inflammation-induced preterm birth and improves newborn outcome
Source: EMBO Mol Med. 2025 Jul 3;17(8):1950–82. doi: 10.1038/s44321-025-00257-9 (PMC12340070; doi:10.1038/s44321-025-00257-9)
Supplement: Supplementary file 1 — Table EV1 [file 44321_2025_257_MOESM1_ESM.docx]

**Table EV1: Primers for mRNA expression analysis**

F= Forward and R= Reverse

| Mouse Primers | |
| --- | --- |
| Il1B -F: 5’-AGATGAAGGGCTGCTTCCAAA-3’ | Il1B-R: 5´-GGAAGGTCCACGGGAAAGAC-3´ |
| Il6-F: 5´-AGACAAAGCCAGAGTCCTTCAG-3´ | Il6-R: 5´-TGCCGAGTAGATCTCAAAGTGA-3´ |
| TNF-F: 5´-TCAGCCGATTTGCTATCTCATA-3´ | TNF-R: 5´-AGTACTTGGGCAGATTGACCTC-3´ |
| Ccl2-F: 5´-CACTCACCTGCTGCTACTCA-3´ | Ccl2-R: 5´-GAGCTTGGTGACAAAAACTACAGC-3´ |
| Ptgs2-F: 5´-TGCCCAGCACTTCACCCATC-3’ | Ptgs2-R: 5´-AGTCCACTCCATGGCCCAGT-3´ |
| Casp1-F: 5´-AGATGCCCACTGCTGATAGG-3´ | Casp1-R: 5´-TTGGCACGATTCTCAGCATA-3´ |
| Oxtr-F: 5´-TGTGTCTCCTTTTGGGACAA-3´ | Oxtr-R: 5´-GGCATTTCAGAATTGGCTGT-3´ |
| Mmp3-F: 5´-GTGACCCCACTCACTTTCTC-3´ | Mmp3-R: 5´-TTGGTACCAGTGACATCCTCT -3´ |
| Ccl3-F: 5´-CCCAGCCAGGTGTCATTTTC-3´ | Ccl3-R: 5´-GTGGCTACTTGGCAGCAAAC-3´ |
| Mmp9-F: 5´-TCAAGGACGGTTGGTACTGG-3´ | Mmp9-R: 5´-CTGACGTGGGTTACCTCTGG-3´ |
| Ptgfr-F : 5´-AGCTGGACTCATCGCAAACA-3´ | Ptgfr-R: 5´-GTGGGCACAAGCCAGAAAAG-3´ |
| Gja1-F : 5´-GCACTTTTCTTTCATTGGGGG-3´ | Gja1-R: 5´-GGGCACCTCTCTTTCACTTA-3´ |
| Mmp1a-F : 5´-CAGGACTTATATGGACCTTCCC-3´ | Mmp1a-R: 5´-TAAATTGAGCTCAGGTTCTGGC-3 |
| **Human Primers** | |
| IL1B-F: 5´-AGCTGGAGAGTGTAGATCCCAA-3´ | IL1B-R: 5´-ACGGGCATGTTTTCTGCTTG-3´ |
| IL6-F: 5´-ACCCCCAGGAGAAGATTCCA-3´ | IL6-R: 5´-CACCAGGCAAGTCTCCTCATT-3´ |
| TNF-F: 5´-AGACCCCTCCCAGATAGATG -3´ | TNF-R: 5´-GCCTGTAGCCCATGTTGTAG-3´ |
