# Supplementary material for: A novel modulator of IL-6R prevents inflammation-induced preterm birth and improves newborn outcome
Source: EMBO Mol Med. 2025 Jul 3;17(8):1950–82. doi: 10.1038/s44321-025-00257-9 (PMC12340070; doi:10.1038/s44321-025-00257-9)
Supplement: Supplementary file 10 — Source data Fig. 8 [file 44321_2025_257_MOESM10_ESM.zip › Figure 7/7G/Identification 2h .pptx]

## Slide 1
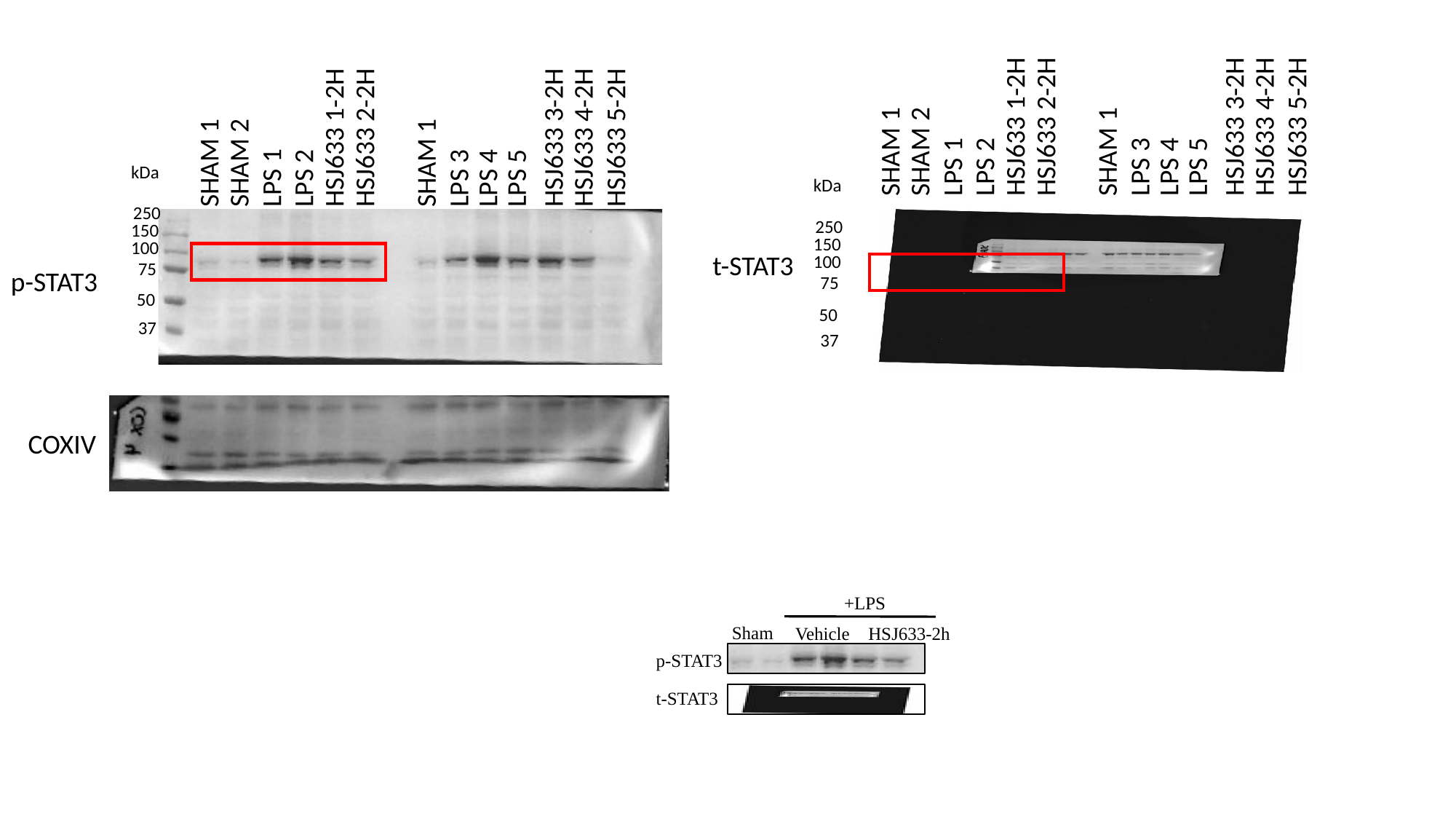

SHAM 1
SHAM 2
LPS 1
LPS 2
HSJ633 1-2H
HSJ633 2-2H
SHAM 1
LPS 3
LPS 4
LPS 5
HSJ633 3-2H
HSJ633 4-2H
HSJ633 5-2H
SHAM 1
SHAM 2
LPS 1
LPS 2
HSJ633 1-2H
HSJ633 2-2H
SHAM 1
LPS 3
LPS 4
LPS 5
HSJ633 3-2H
HSJ633 4-2H
HSJ633 5-2H
kDa
250
150
100
75
50
37
kDa
250
150
100
75
50
37
t-STAT3
p-STAT3
COXIV
+LPS
Sham
HSJ633-2h
p-STAT3
t-STAT3
Vehicle

## Slide 2
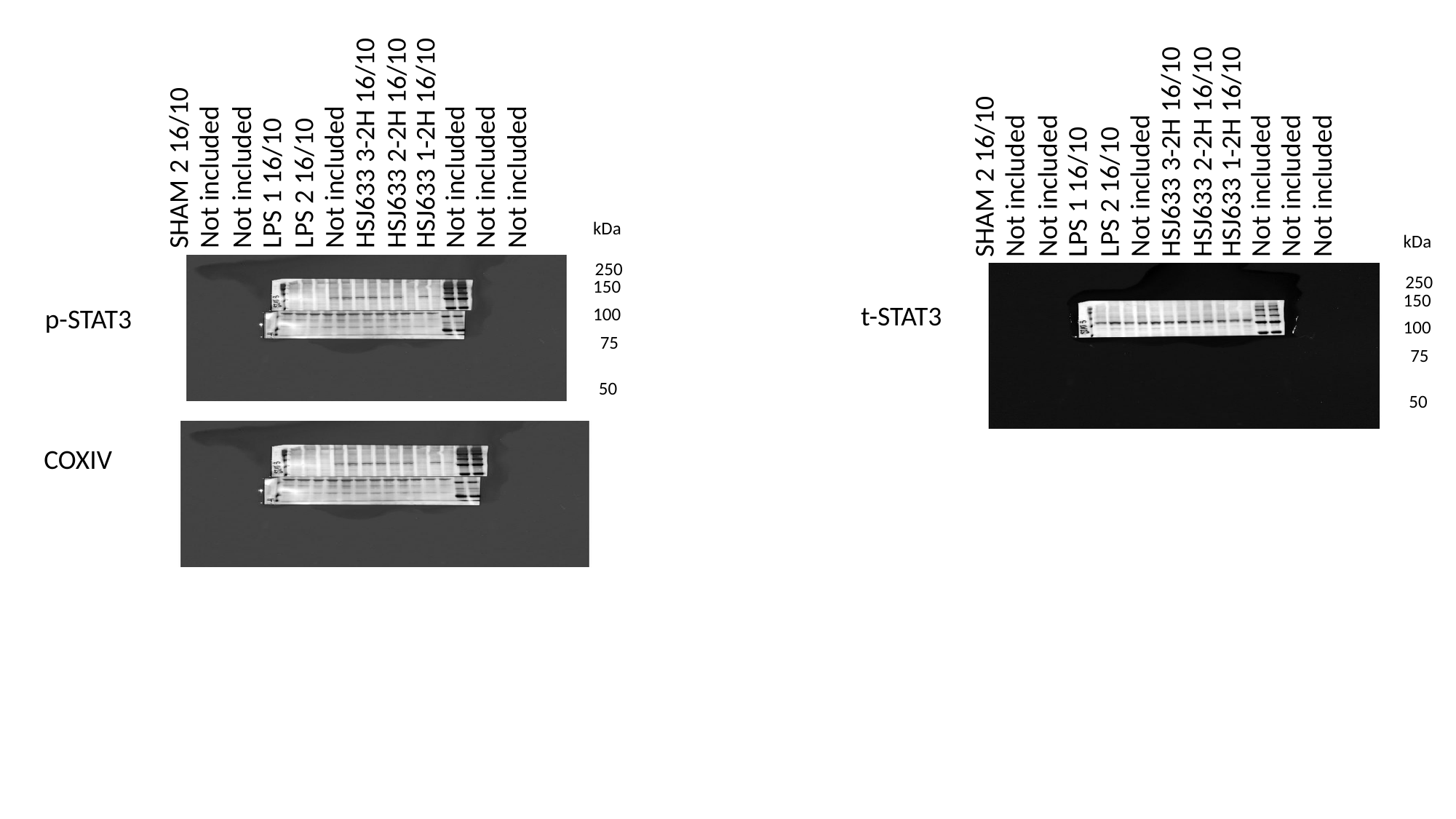

Not included
Not included
Not included
Not included
Not included
Not included
HSJ633 3-2H 16/10
HSJ633 1-2H 16/10
LPS 1 16/10
LPS 2 16/10
SHAM 2 16/10
HSJ633 2-2H 16/10
Not included
Not included
Not included
Not included
Not included
Not included
HSJ633 3-2H 16/10
HSJ633 1-2H 16/10
LPS 1 16/10
LPS 2 16/10
SHAM 2 16/10
HSJ633 2-2H 16/10
kDa
250
150
100
75
50
kDa
250
150
100
75
50
t-STAT3
p-STAT3
COXIV
