# Supplementary material for: A novel modulator of IL-6R prevents inflammation-induced preterm birth and improves newborn outcome
Source: EMBO Mol Med. 2025 Jul 3;17(8):1950–82. doi: 10.1038/s44321-025-00257-9 (PMC12340070; doi:10.1038/s44321-025-00257-9)
Supplement: Supplementary file 10 — Source data Fig. 8 [file 44321_2025_257_MOESM10_ESM.zip › Figure 7/7F/identification prevention .pptx]

## Slide 1
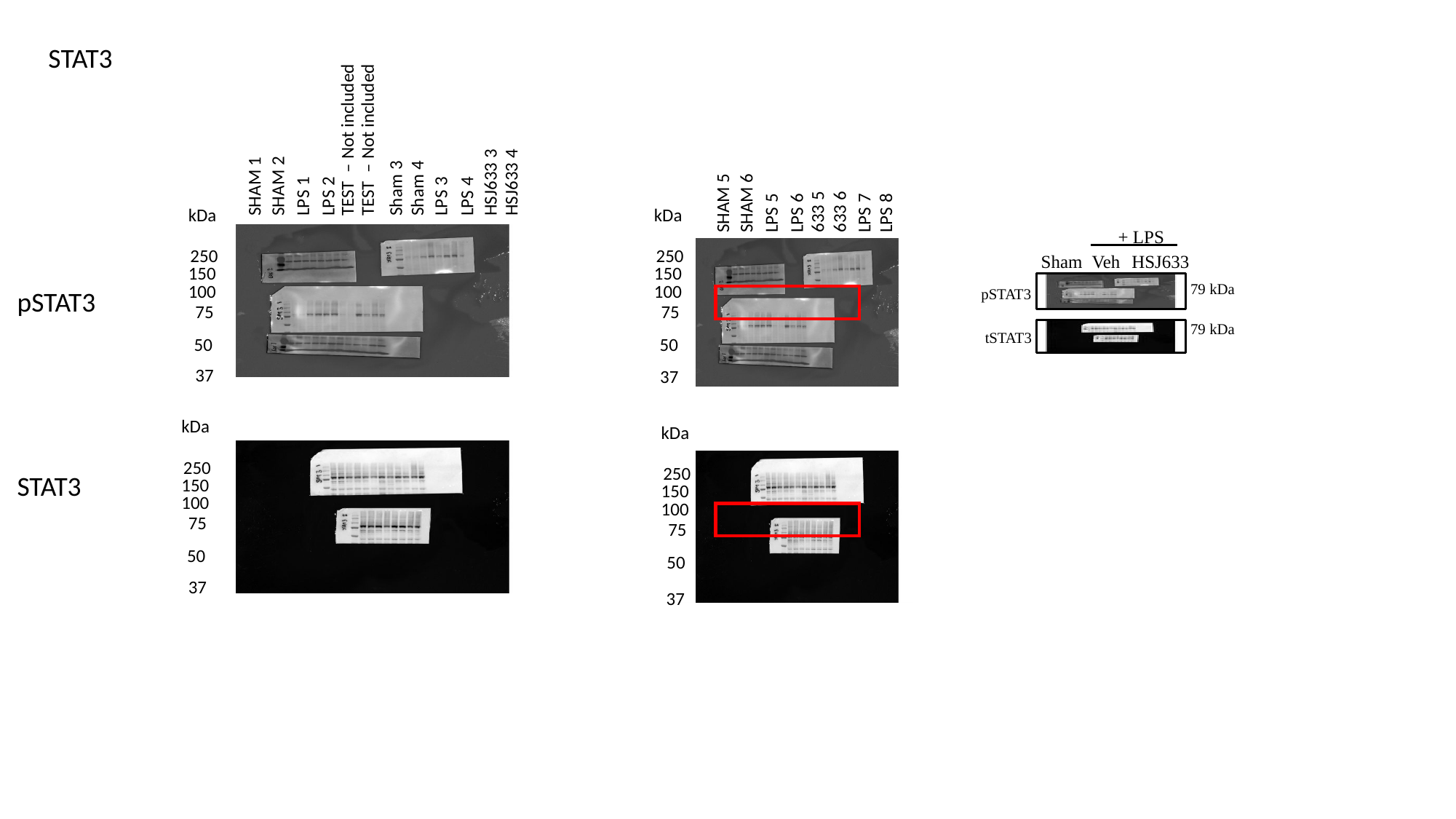

STAT3
TEST – Not included
SHAM 1
SHAM 2
LPS 2
Sham 3
Sham 4
LPS 3
LPS 4
HSJ633 4
LPS 1
HSJ633 3
TEST – Not included
SHAM 5
SHAM 6
LPS 6
633 5
633 6
LPS 7
LPS 8
LPS 5
kDa
250
150
100
75
50
37
kDa
250
150
100
75
50
+ LPS
Sham
HSJ633
Veh
79 kDa
pSTAT3
79 kDa
 tSTAT3
pSTAT3
37
kDa
250
150
100
75
50
37
kDa
250
150
100
75
50
STAT3
37
