# Supplementary material for: A novel modulator of IL-6R prevents inflammation-induced preterm birth and improves newborn outcome
Source: EMBO Mol Med. 2025 Jul 3;17(8):1950–82. doi: 10.1038/s44321-025-00257-9 (PMC12340070; doi:10.1038/s44321-025-00257-9)
Supplement: Supplementary file 10 — Source data Fig. 8 [file 44321_2025_257_MOESM10_ESM.zip › Figure 7/7A/p38 identification .pptx]

## Slide 1
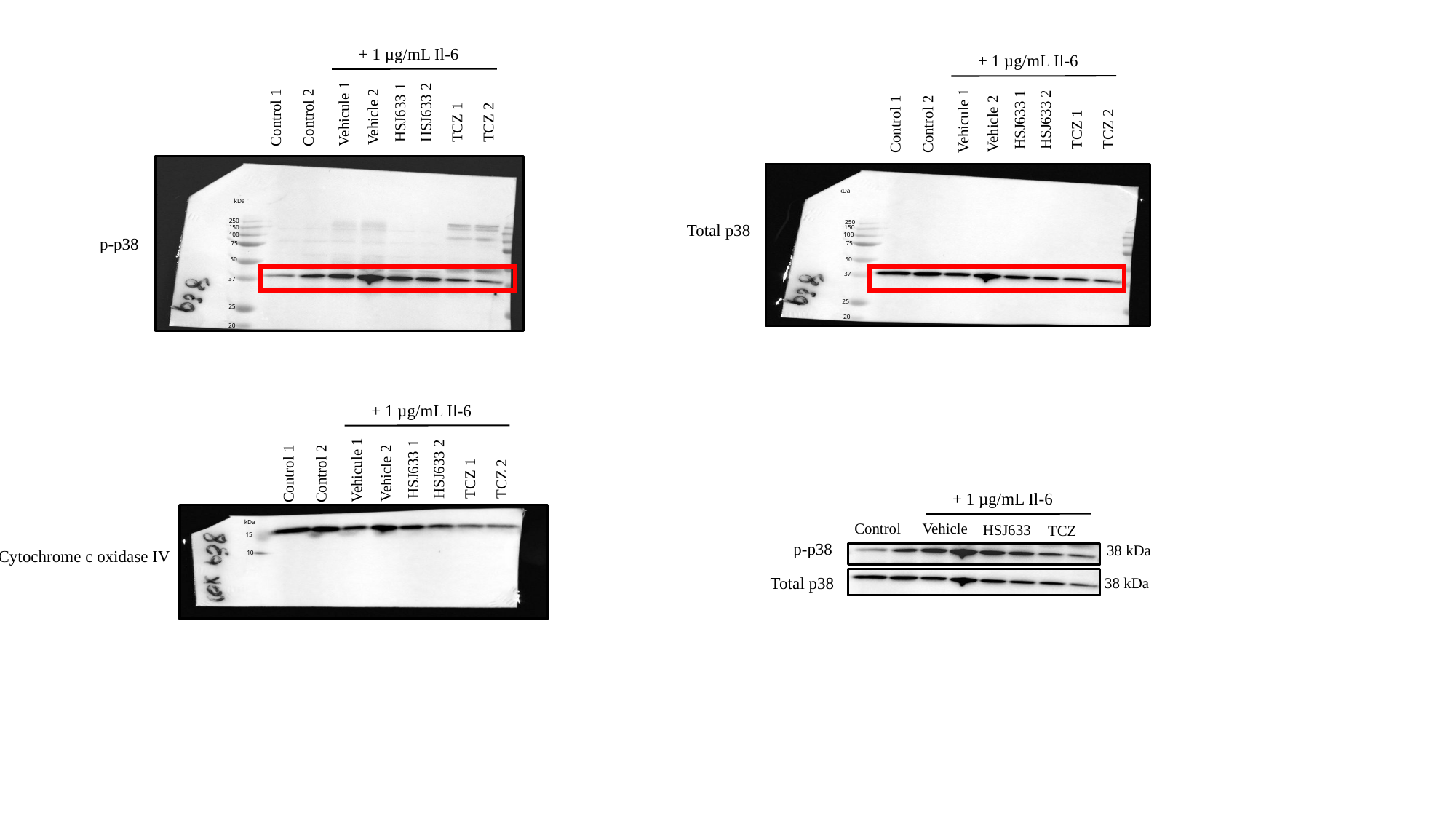

Vehicle 2
Vehicule 1
 HSJ633 1
 HSJ633 2
Control 1
Control 2
 TCZ 2
 TCZ 1
+ 1 µg/mL Il-6
Vehicle 2
Vehicule 1
 HSJ633 1
 HSJ633 2
Control 1
Control 2
 TCZ 2
 TCZ 1
+ 1 µg/mL Il-6
kDa
250
150
100
75
50
37
25
20
kDa
250
150
100
75
50
37
25
20
Total p38
p-p38
Vehicle 2
Vehicule 1
 HSJ633 1
 HSJ633 2
Control 1
Control 2
 TCZ 2
 TCZ 1
+ 1 µg/mL Il-6
+ 1 µg/mL Il-6
Vehicle
Control
 HSJ633
TCZ
p-p38
38 kDa
Total p38
38 kDa
kDa
15
10
Cytochrome c oxidase IV
