# Supplementary figures and images for: A novel modulator of IL-6R prevents inflammation-induced preterm birth and improves newborn outcome
Source: EMBO Mol Med. 2025 Jul 3;17(8):1950–82. doi: 10.1038/s44321-025-00257-9 (PMC12340070; doi:10.1038/s44321-025-00257-9)

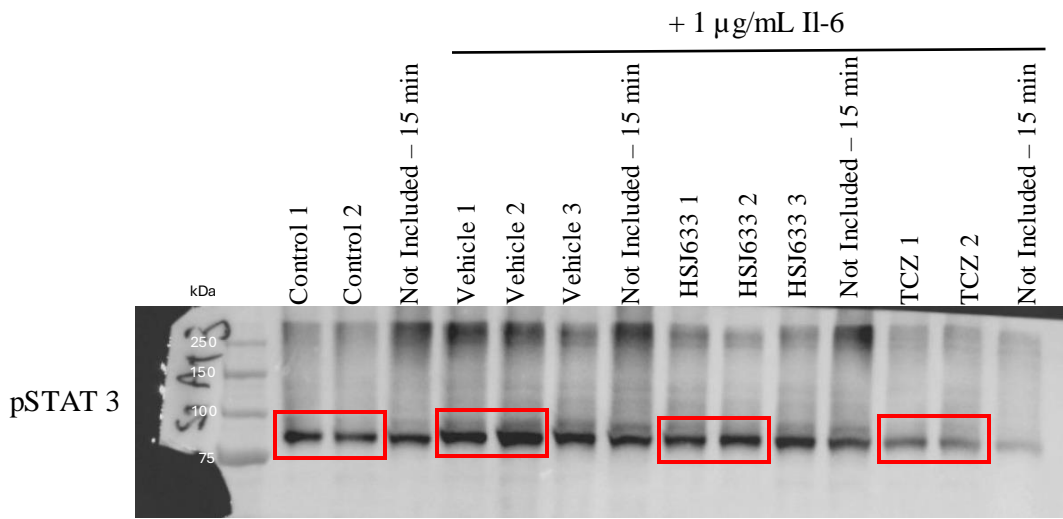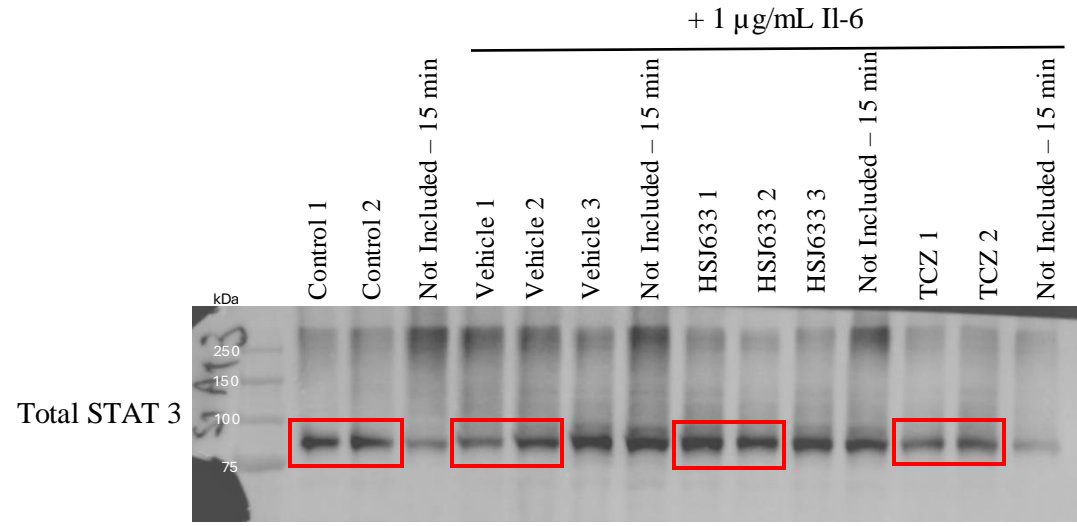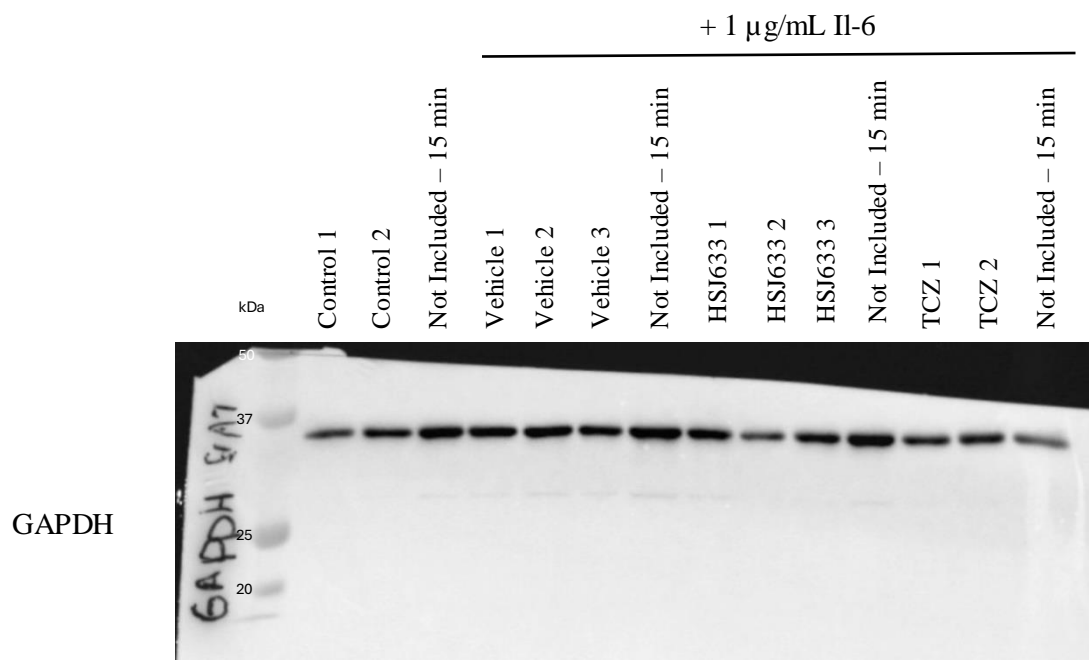

Supplement: Supplementary file 10 — Source data Fig. 8 [file 44321_2025_257_MOESM10_ESM.zip › Figure 7/7D/identification stat3 .pdf]

pAKT

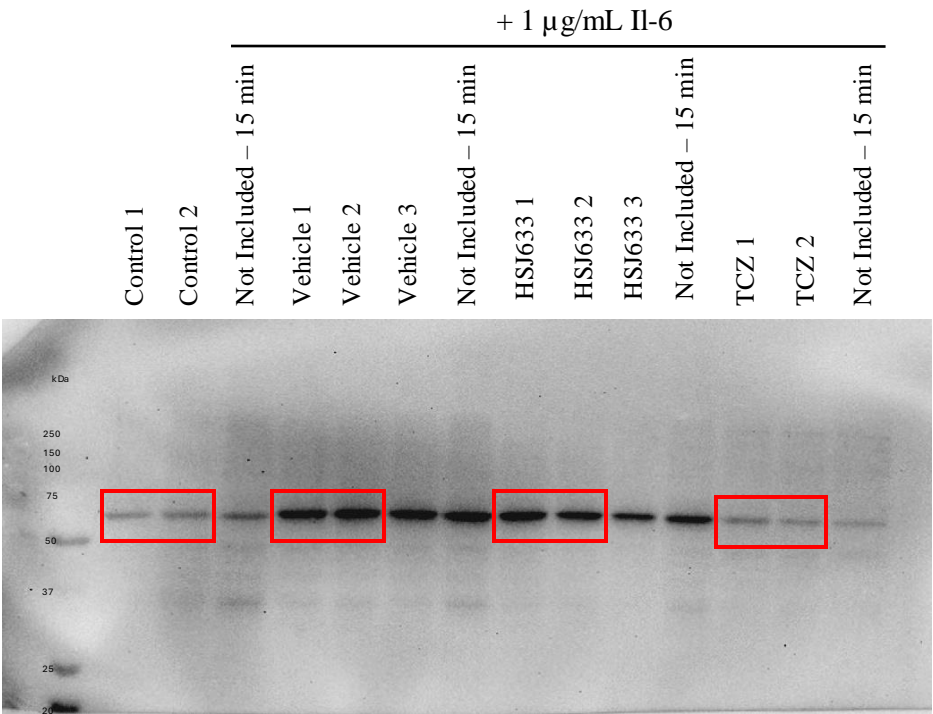

Total AKT

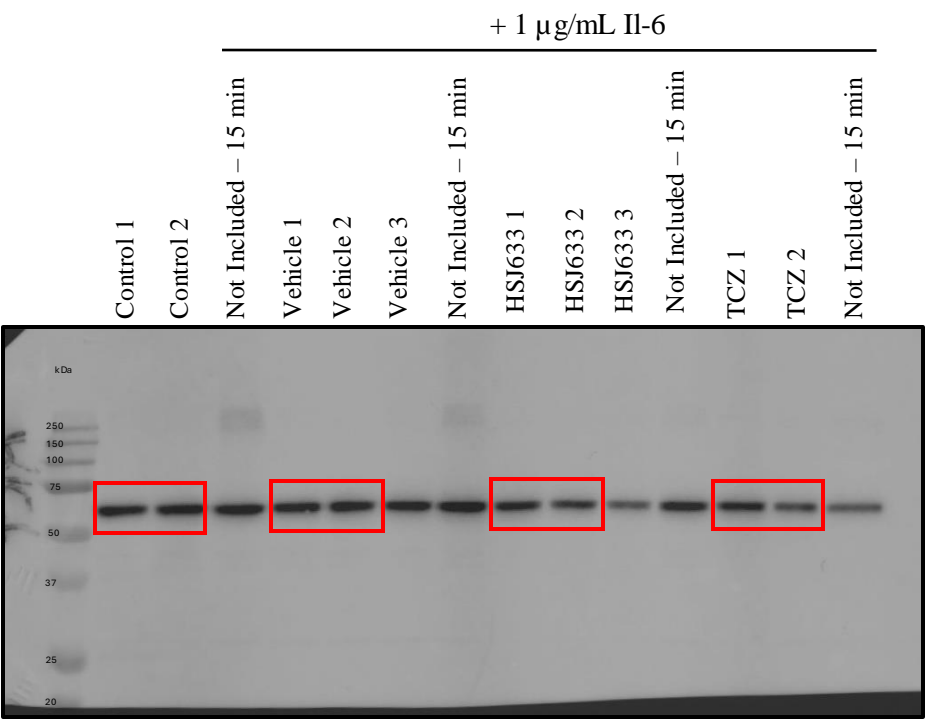

Cytochrome c oxidase IV

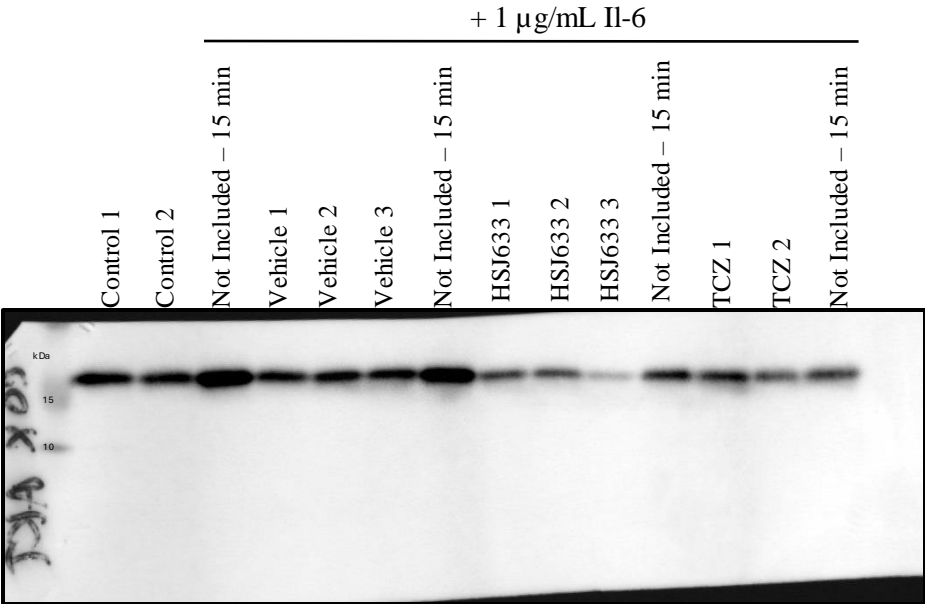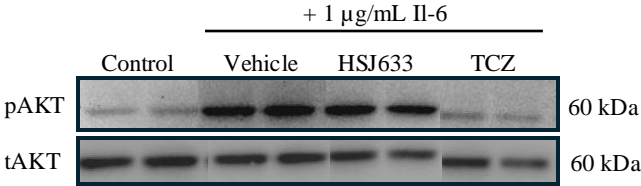

Supplement: Supplementary file 10 — Source data Fig. 8 [file 44321_2025_257_MOESM10_ESM.zip › Figure 7/7C/AKT Identification .pdf]

pERK 1/2

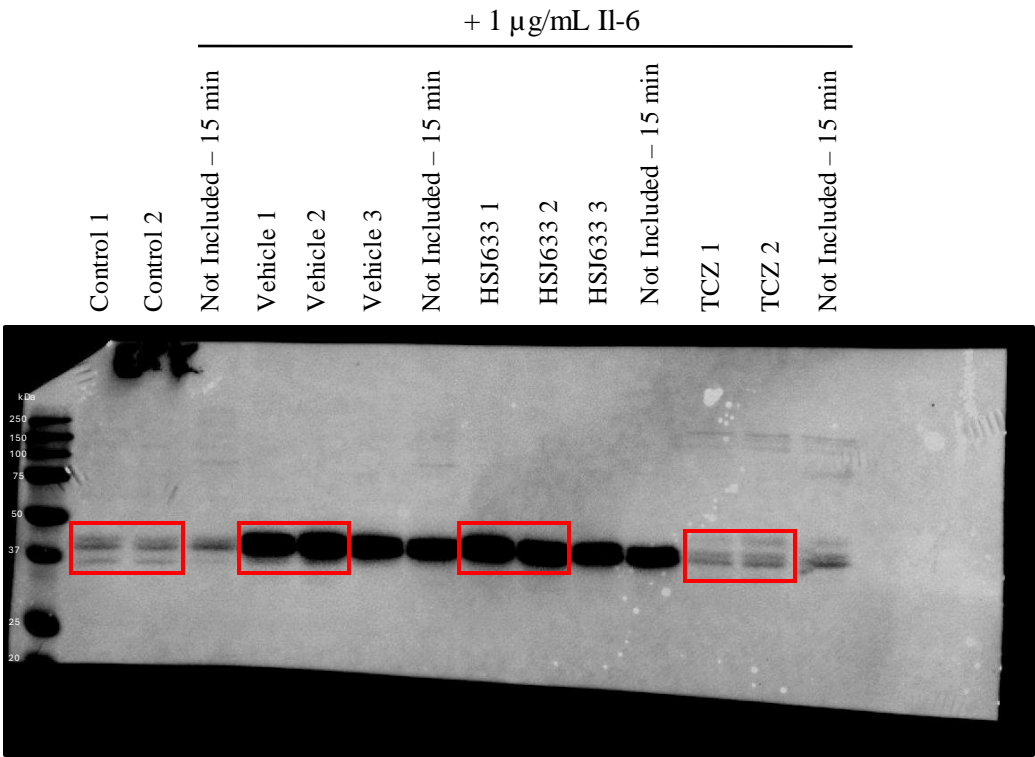

Total ERK 1/2

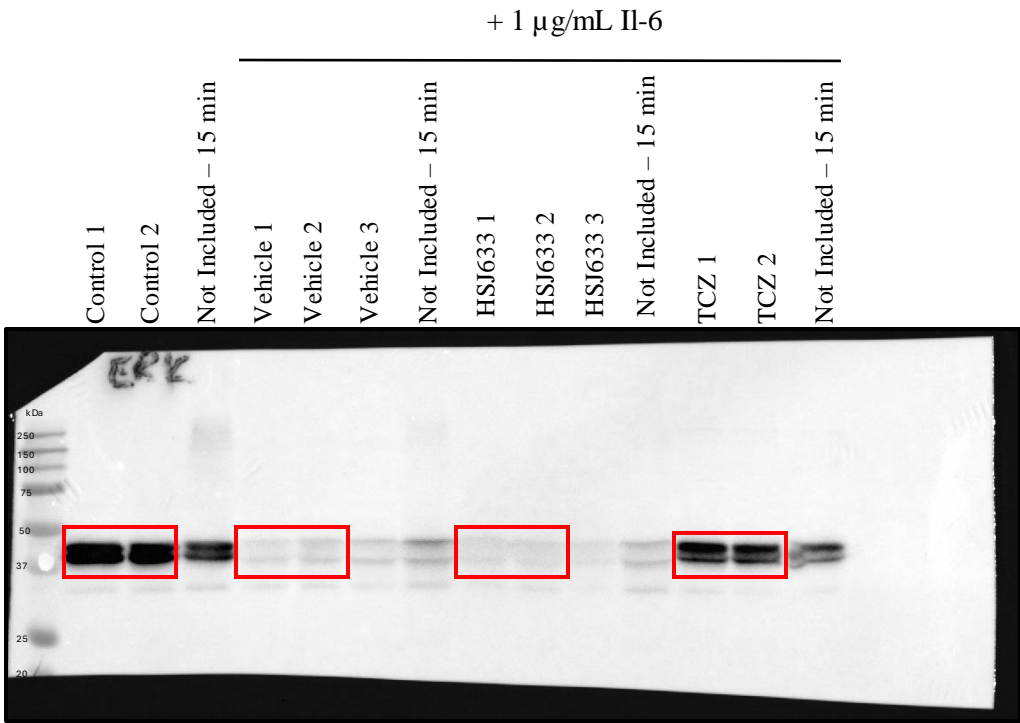

Cytochrome c oxidase IV

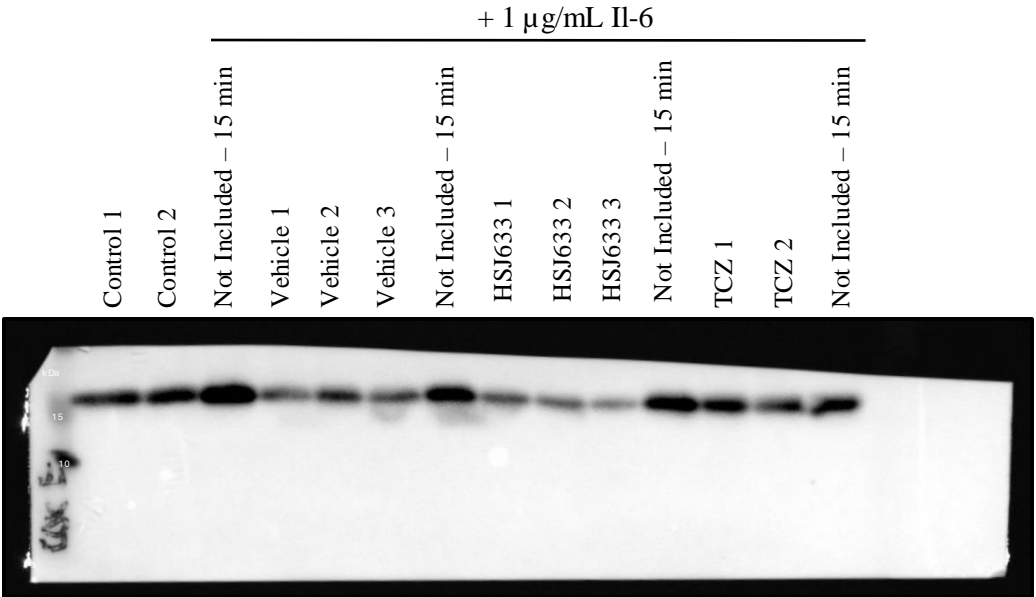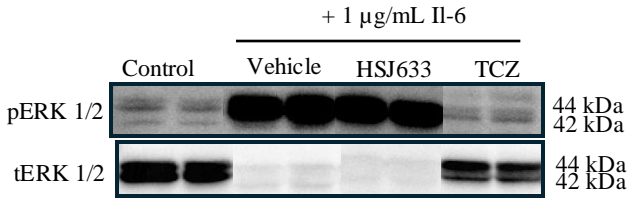

Supplement: Supplementary file 10 — Source data Fig. 8 [file 44321_2025_257_MOESM10_ESM.zip › Figure 7/7B/ERK identification .pdf]

p-STAT3

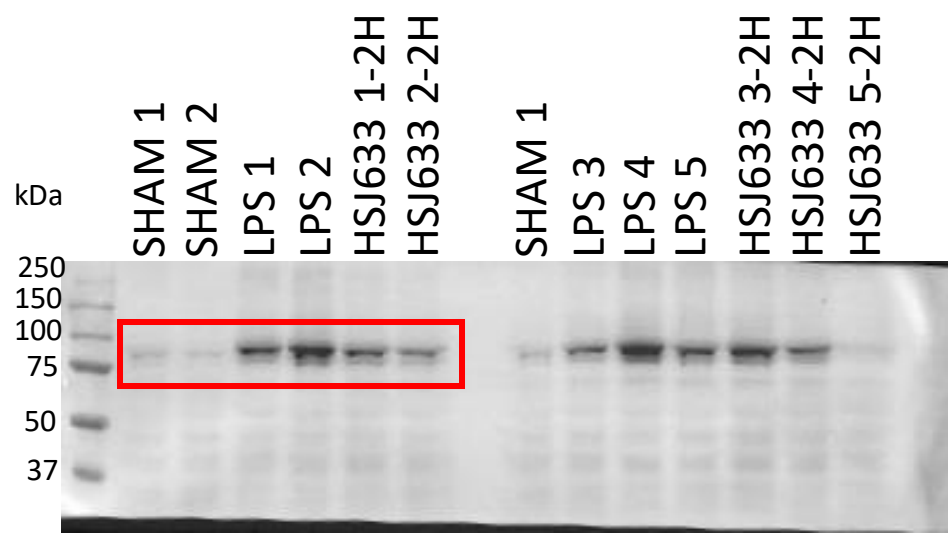

COXIV

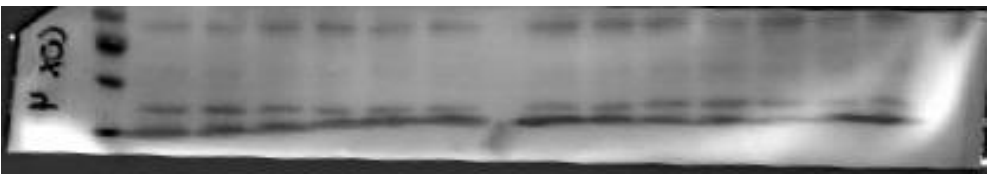

t-STAT3

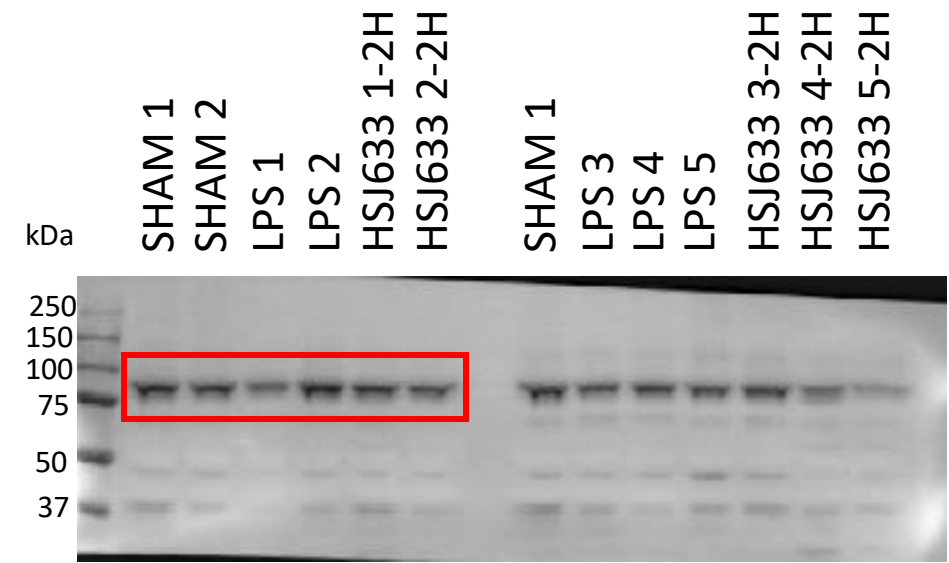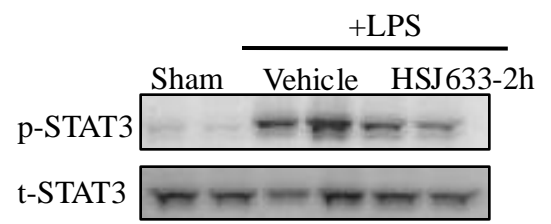

p-STAT3

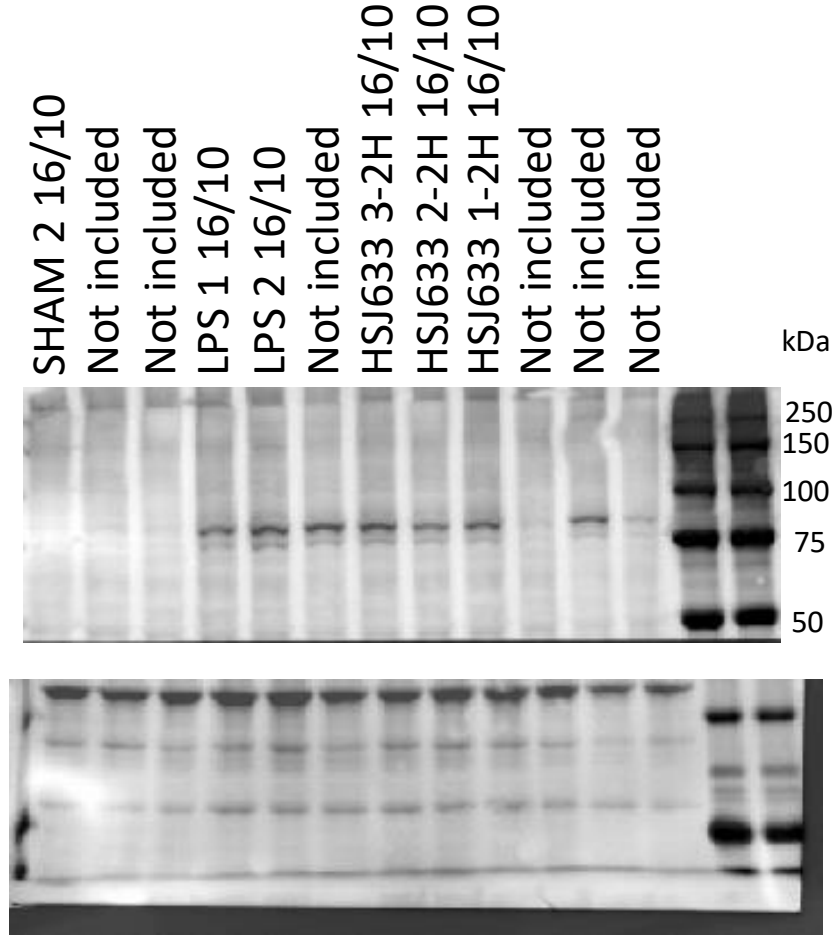

t-STAT3

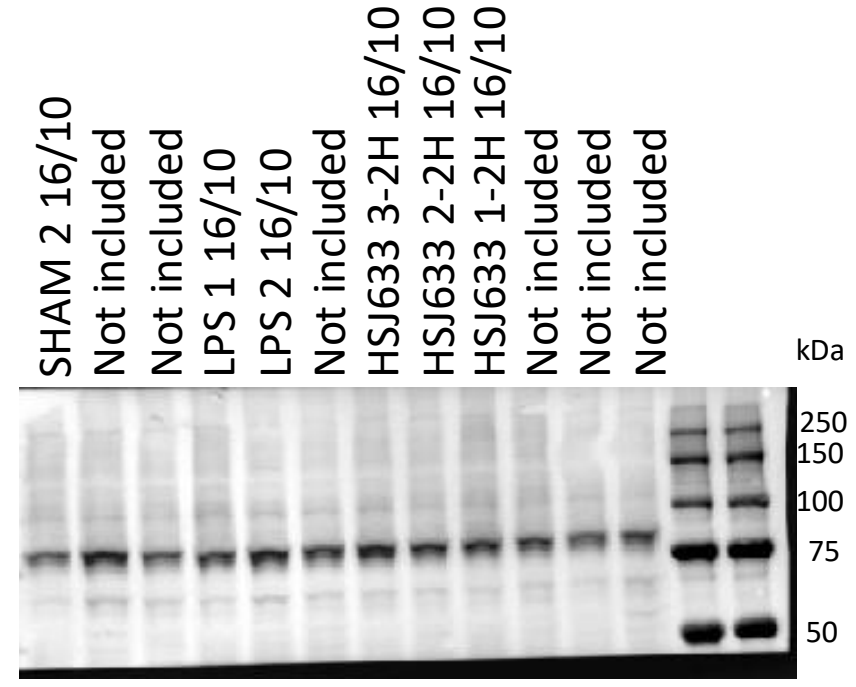

Supplement: Supplementary file 10 — Source data Fig. 8 [file 44321_2025_257_MOESM10_ESM.zip › Figure 7/7G/Identification 2h .pdf]

STAT3

pSTAT3

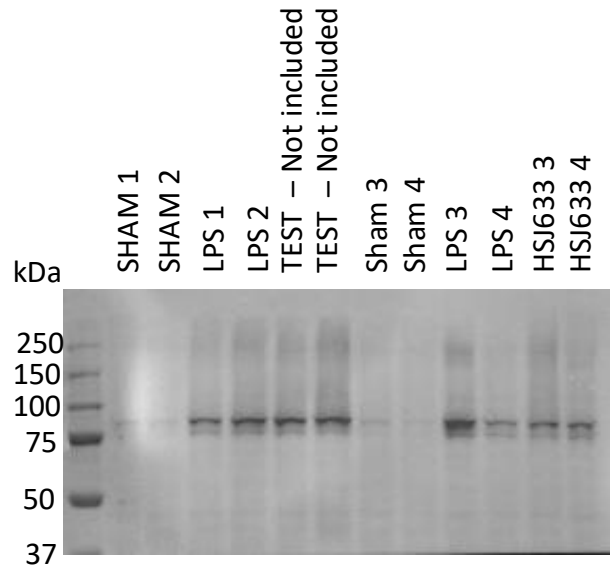

STAT3

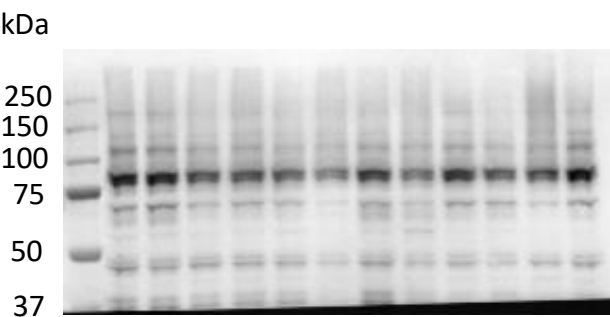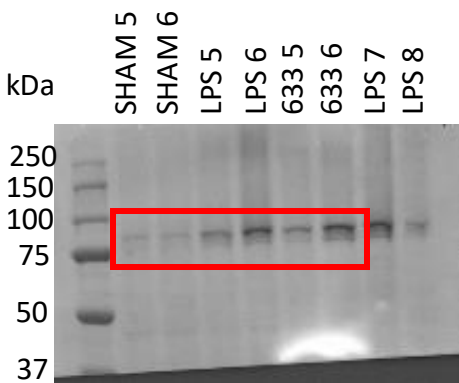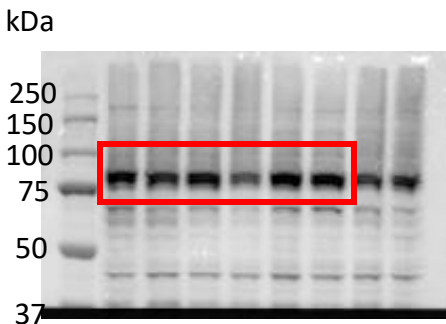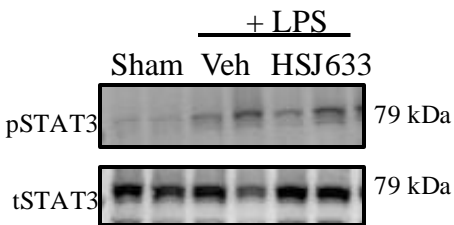

Supplement: Supplementary file 10 — Source data Fig. 8 [file 44321_2025_257_MOESM10_ESM.zip › Figure 7/7F/identification prevention .pdf]

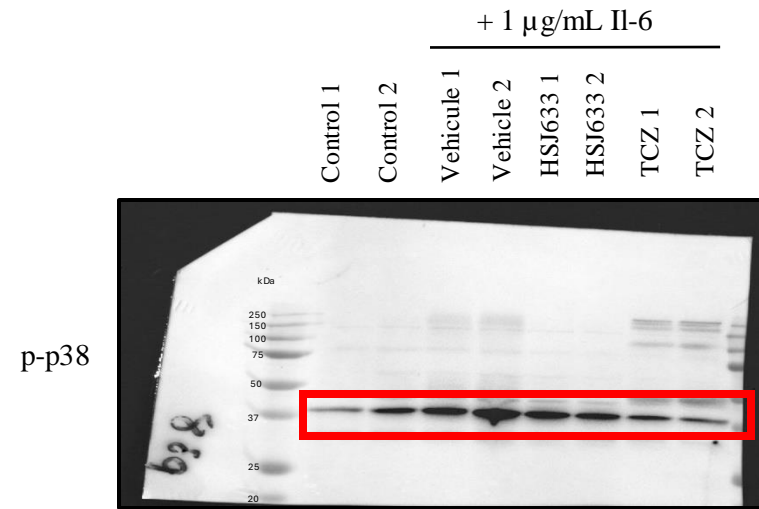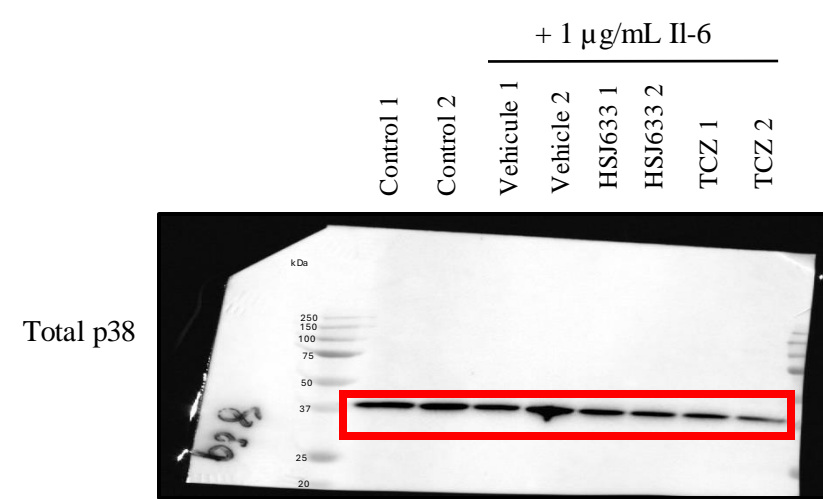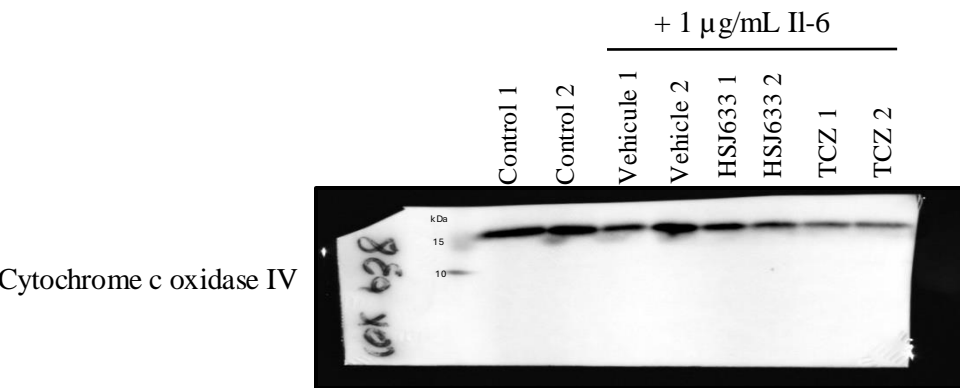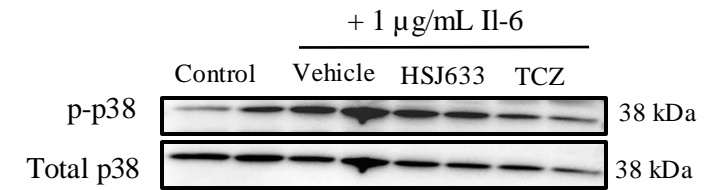

Supplement: Supplementary file 10 — Source data Fig. 8 [file 44321_2025_257_MOESM10_ESM.zip › Figure 7/7A/p38 identification .pdf]
