# Supplementary material for: A novel modulator of IL-6R prevents inflammation-induced preterm birth and improves newborn outcome
Source: EMBO Mol Med. 2025 Jul 3;17(8):1950–82. doi: 10.1038/s44321-025-00257-9 (PMC12340070; doi:10.1038/s44321-025-00257-9)
Supplement: Supplementary file 11 — Expanded View Figures [file 44321_2025_257_MOESM11_ESM.pdf]

Expanded View Figures

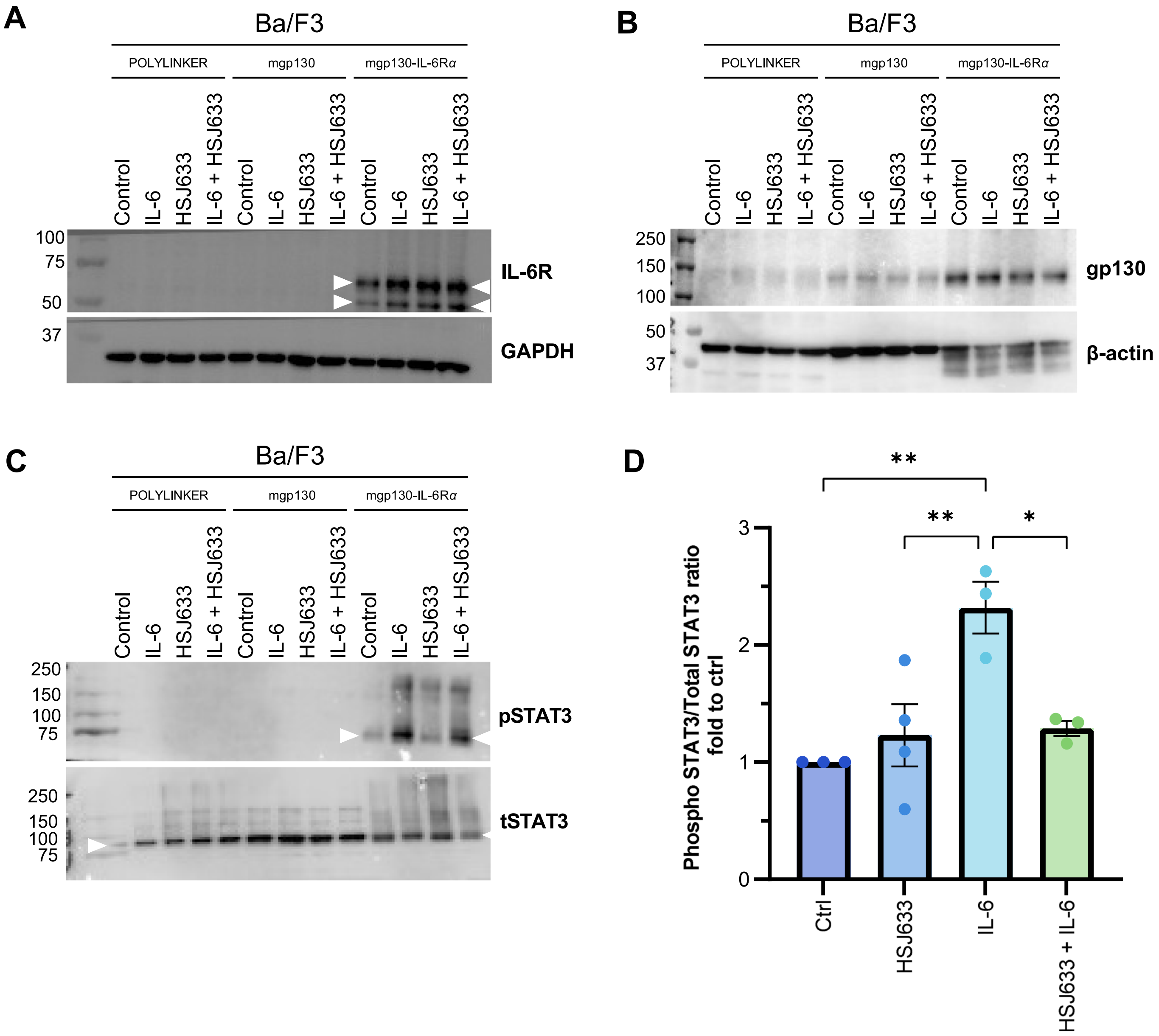

Figure EV1. HSJ633 reduces STAT3 activation in murine Ba/F3-mgp130-IL-6Rα cells but not in murine cells without IL-6Rα.

Representative Western blot displaying the levels of IL-6Rα, gp130 and STAT3 in Ba/F3-polylinker, Ba/F3-mgp130, and Ba/F3-mgp130-IL-6Rα cells treated with IL-6 (1.92 nM), HSJ633 (0.1 mM) and HSJ633 + IL-6. (**A**) Western blot image showing IL-6Rα and GAPDH bands. (**B**) Western blot image showing gp130 and β-actin bands. (**C**) Western blot image showing phospho-STAT3 and total STAT3 bands. (**D**) Quantification of STAT3 activation in Ba/F3-mgp130-IL-6Rα, presented as mean ± SEM of 3-4 samples per group. Densitometric analysis of the bands indicates a significant reduction in STAT3 activation in HSJ633 + IL-6-treated cells relative to IL-6. One-way ANOVA with Dunnett's multiple comparisons test compared to IL-6. *$P < 0.05$, **$P < 0.01$.

 © The Author(s)

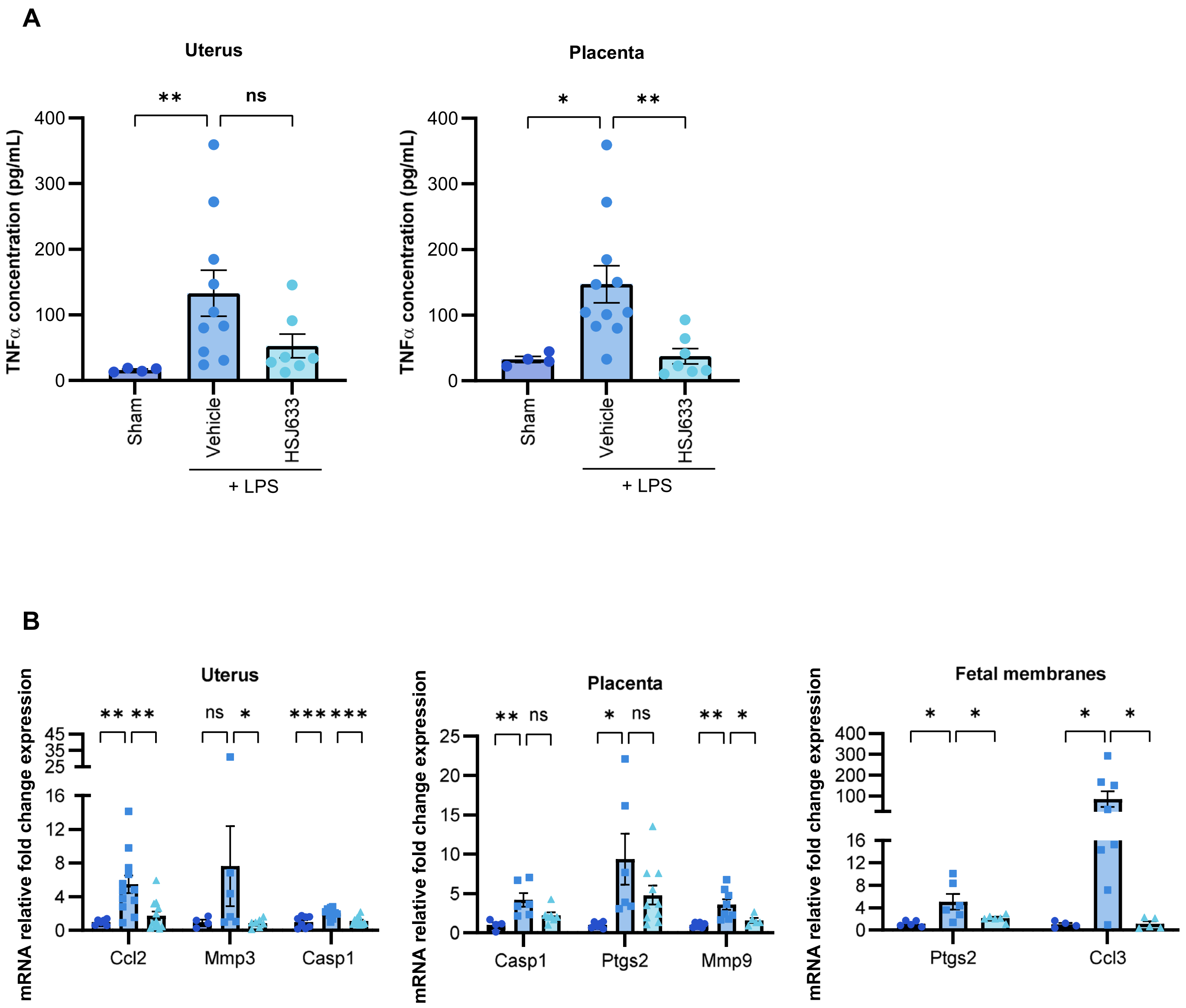

Figure EV2. Prophylactic administration of HSJ633 reduces inflammation in reproductive tissues.

(**A**) TNF-α concentrations (quantified by ELISA) in the uterus and placenta on GD17.25. Values are mean ± SEM of 4–10 dams per group and one-way ANOVA with Dunnett's multiple comparisons test compared to LPS + vehicle group was performed for the placenta. Values are mean ± SEM of 4–11 dams per group and Kruskal–Wallis test with Dunn's multiple comparisons test compared to the LPS + vehicle group was performed for the uterus. *$P < 0.05$, **$P < 0.01$. (**B**) mRNA expression on GD17.25 in the uterus, placenta, and fetal membranes of various genes involved in PTB (normalized to Actb). Values are mean ± SEM of 4–12 dams per group for the uterus, 4–10 dams per group in the placenta and 4–8 dams per group for fetal membranes. *$P < 0.05$, **$P < 0.01$, ***$P < 0.001$,

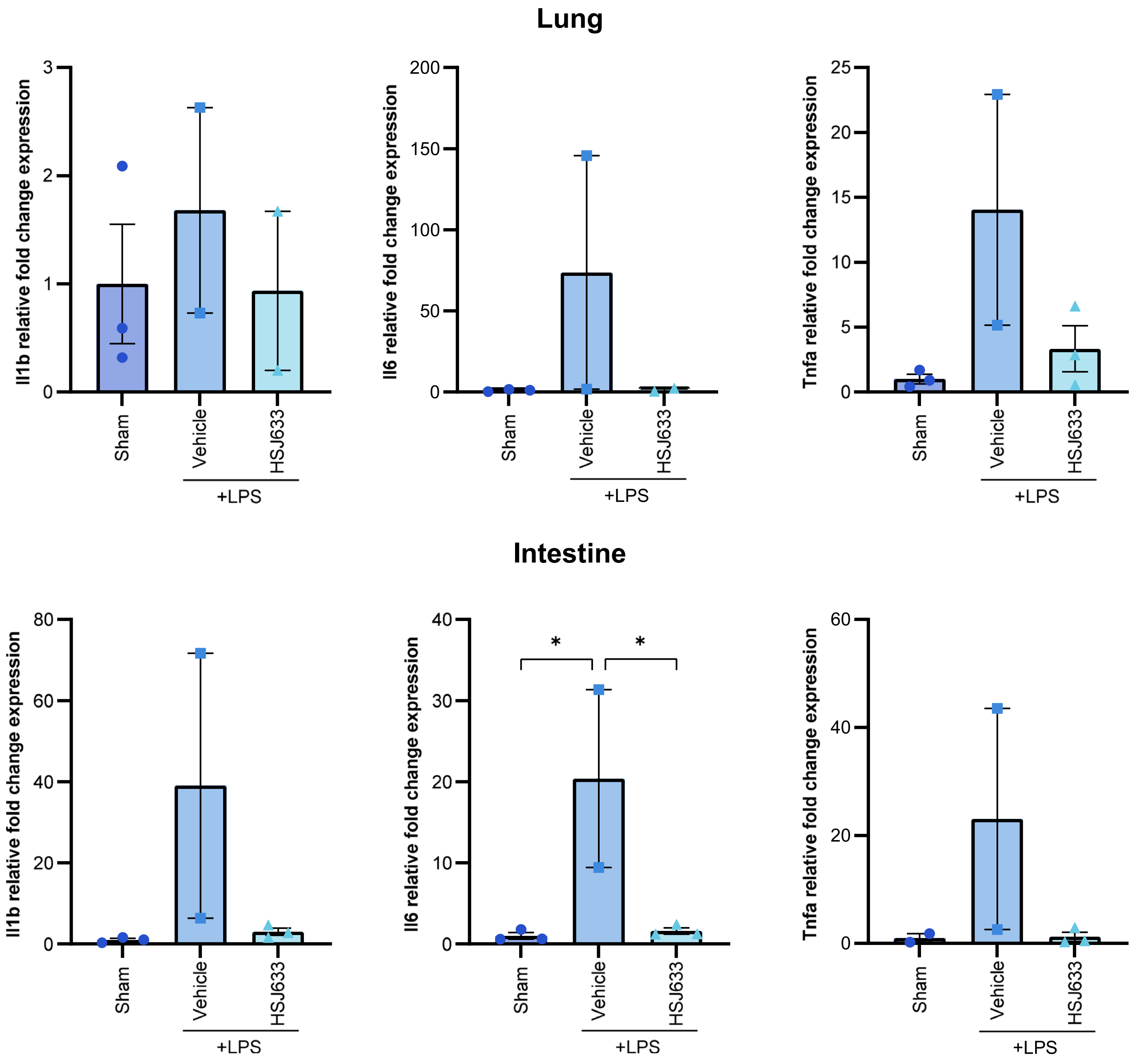

Figure EV3. LPS-induced inflammatory cytokines in fetal organs.

mRNA expression (determined by RT-qPCR) of IL-1β, IL-6 and TNF-α in murine lung and intestine collected on GD17.25 after LPS induction (normalized to Actb). Values are mean ± SEM of 2–5 samples per group. One-way ANOVA with Dunnett's multiple comparisons test compared to LPS + vehicle group. *$P < 0.05$.

 © The Author(s)

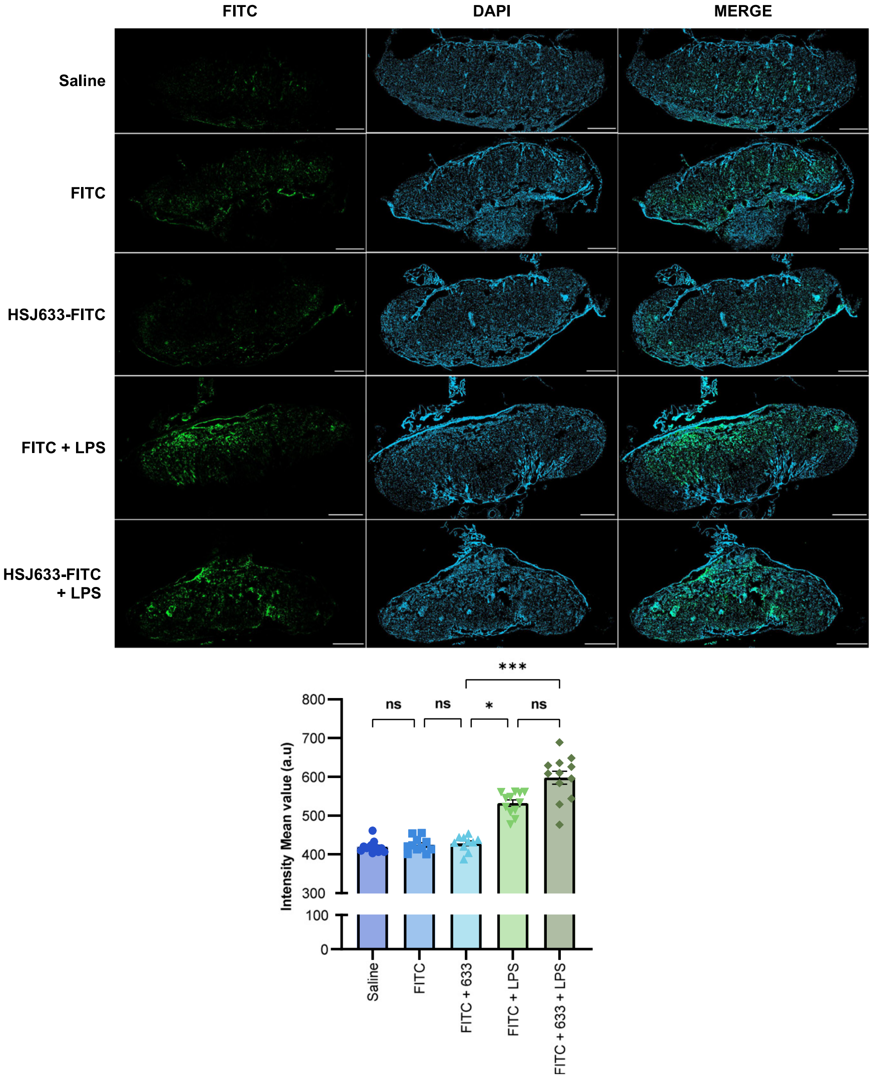

Figure EV4. Distribution of HSJ633 to fetal placental compartment.

Biodistribution of prophylactic HSJ633-FITC 4 h post subcutaneous injection in pregnant mice on GD17 in the placenta. Green fluorescence was quantified. Values are mean ± SEM of 11–12 samples per group. 1000 µm scale bars. *$P < 0.05$, ***$P < 0.001$ by the Kruskal–Wallis test with Dunn's multiple comparisons test compared to the LPS + vehicle group.

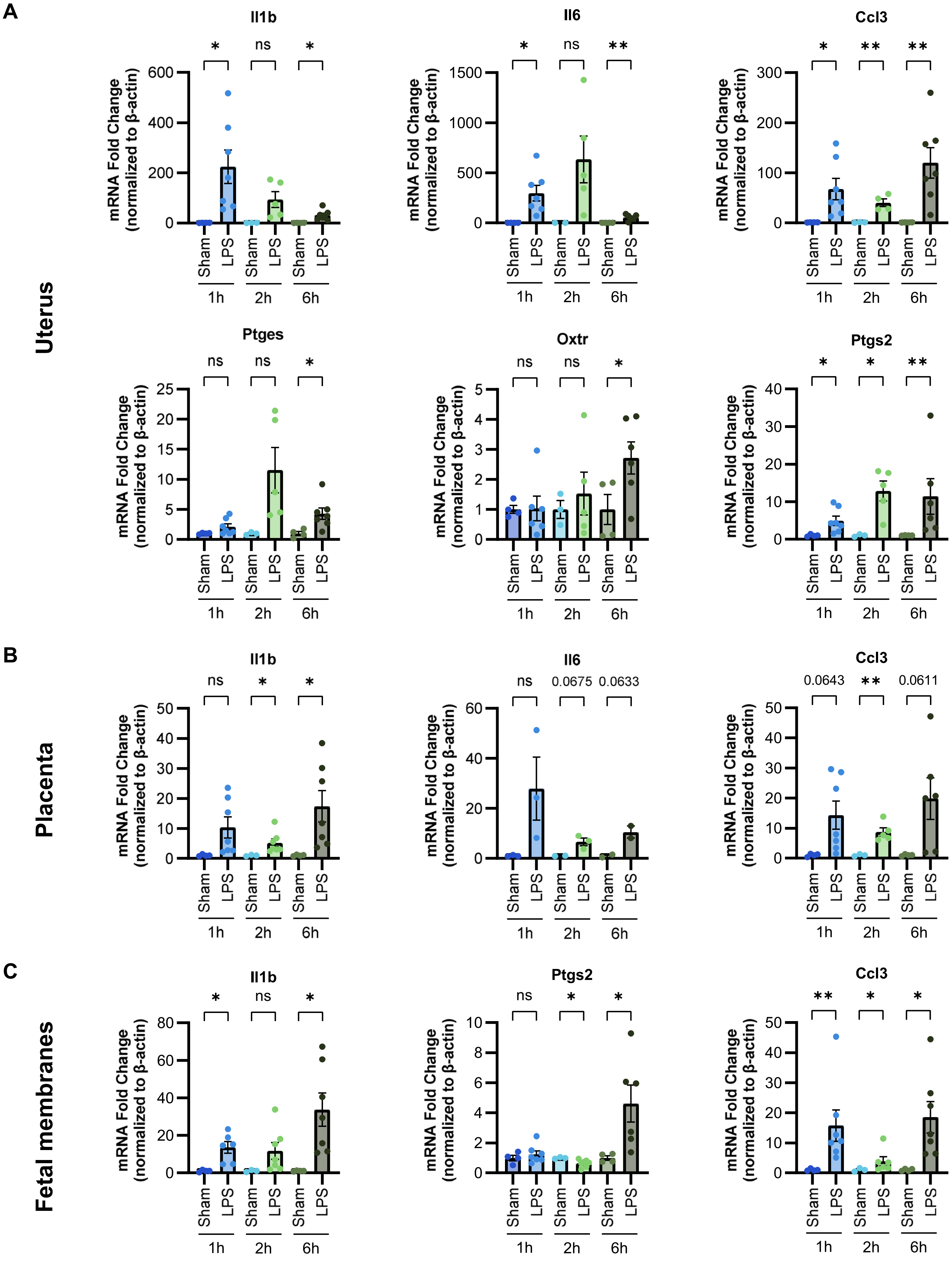
A
Uterus
Il1b
Il6
Ccl3
Ptges
Oxtr
Ptgs2
B
Placenta
Il1b
Il6
Ccl3
0.0675
0.0633
0.0643
0.0611
C
Fetal membranes
Il1b
Ptgs2
Ccl3
ns
mRNA Fold Change (normalized to β-actin)
Sham
LPS
1h
2h
6h

 © The Author(s)

Figure EV5. LPS induces an inflammatory response as early as 1 h post-administration in gestational tissues.

(**A–C**) mRNA expression (determined by RT-qPCR) of IL-1β, IL-6, Ccl3, Ptgs2, Ptges, and Oxtr in the uterus, placenta, and fetal membranes collected 1 h, 2 h and 6 h after LPS injection on GD16 (normalized to Actb). Values are mean ± SEM of 3–7 samples per group. Unpaired *T* test compared to sham group at each time point with Mann–Whitney comparison when data were not distributed normally. *$P < 0.05$, **$P < 0.01$.

© The Author(s)

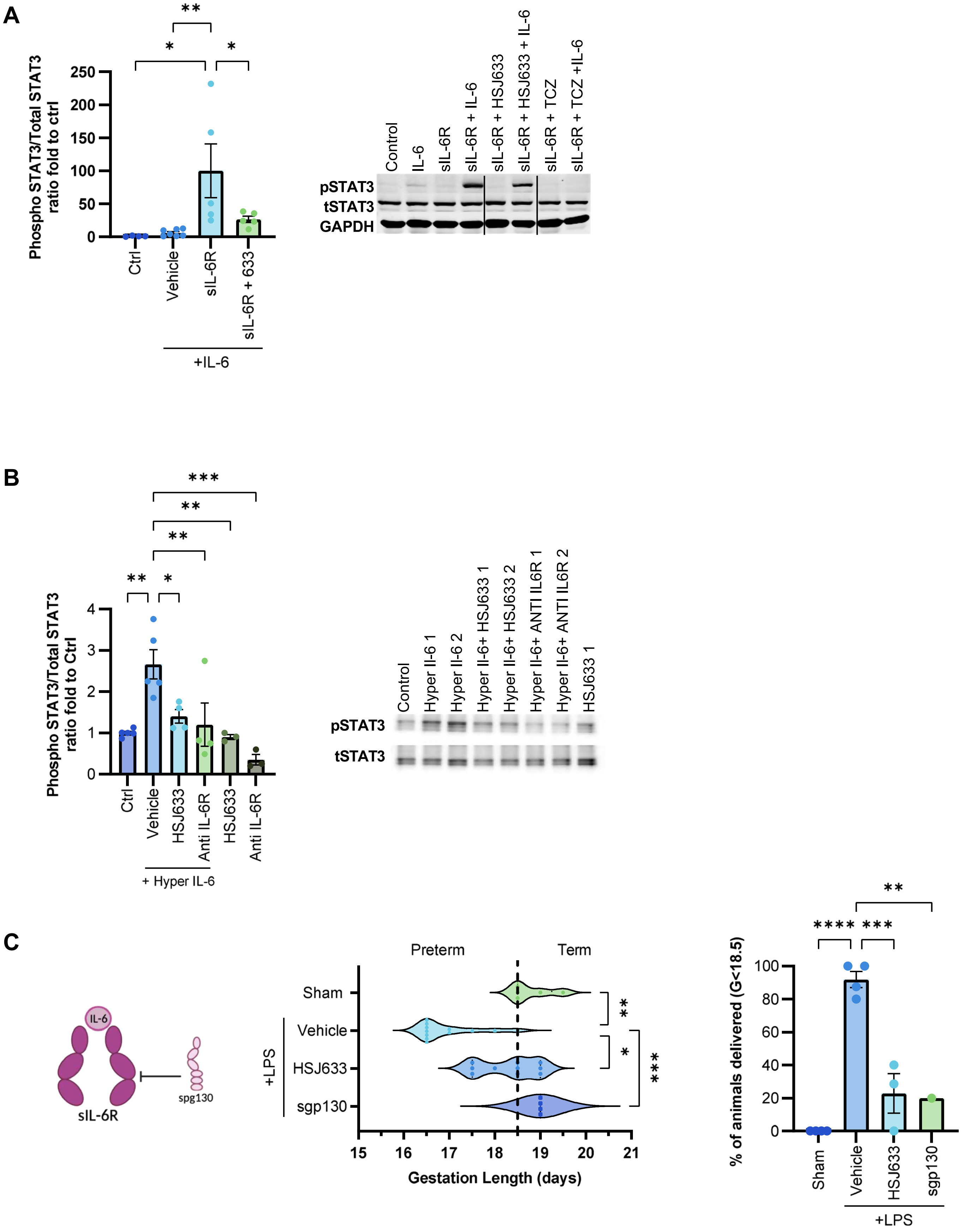
A
Phospho STAT3/Total STAT3 ratio fold to ctrl
Ctrl
Vehicle
sIL-6R
sIL-6R + 633
+IL-6
Control
IL-6
sIL-6R
sIL-6R + IL-6
sIL-6R + HSJ633
sIL-6R + HSJ633 + IL-6
sIL-6R + TCZ
sIL-6R + TCZ +IL-6
pSTAT3
tSTAT3
GAPDH
B
Phospho STAT3/Total STAT3 ratio fold to Ctrl
Ctrl
Vehicle
HSJ633
Anti IL-6R
HSJ633
Anti IL-6R
+ Hyper IL-6
Control
Hyper Il-6 1
Hyper Il-6 2
Hyper Il-6+ HSJ633 1
Hyper Il-6+ HSJ633 2
Hyper Il-6+ ANTI IL6R 1
Hyper Il-6+ ANTI IL6R 2
HSJ633 1
pSTAT3
tSTAT3
C
IL-6
sIL-6R
spg130
Preterm
Term
Sham
Vehicle
HSJ633
sgp130
+LPS
Gestation Length (days)
% of animals delivered (G<18.5)
Sham
Vehicle
HSJ633
sgp130
+LPS

 © The Author(s)

Figure EV6. Importance of soluble IL-6R in human amniotic epithelial cells and in Ba/F3-mgp130-IL-6Rα IL-6-triggered signaling and in LPS-induced PTB.

(**A**) Amniotic epithelial cells were stimulated with IL-6 and/or sIL-6R (100 ng/mL for both) in absence or presence of HSJ633 (1 μM), TCZ (3.44 μM), or vehicle. Membranes were blotted for phospho-STAT3 and normalized to total STAT3. Representative Western blot gel on the right and compiled histogram data on the left. Values are mean ± SEM of 4–7 samples per group. One-way ANOVA with Dunnett's multiple comparisons test compared to sIL-6R + IL-6 group. (**B**) Quantification of STAT3 activation in Ba/F3-mgp130-IL-6Rα treated with Hyper IL-6 (100 ng/mL), HSJ633 (0.1 mM) and Anti-IL-6R (200 μg/mL). Representative Western blot displaying the levels of STAT3 in Ba/F3-mgp130-IL-6Rα cells treated with hyper IL-6. Quantification of STAT3 activation presented as mean ± SEM of 3–5 samples per group. Densitometric analysis of the bands indicates a significant reduction in STAT3 activation in HSJ633+Hyper IL-6-treated cells relative to Hyper IL-6. One-way ANOVA with Dunnett's multiple comparisons test compared to Hyper IL-6. *$P < 0.05$, **$P < 0.01$, ***$P < 0.001$. (**C**) Gestational length (left panel), data are presented as mean ± SEM of 4–12 dams per group and Kruskal–Wallis test with Dunn's multiple comparisons test compared to the LPS + vehicle group for gestation length. Rate of prematurity (born on GD < 18.5; right panel) in mice treated with soluble gp130 (sgp130) injected s.c. on GD16. Data are presented as mean ± SEM of 1–4 different experiments with 2 to 8 dams per group per experiment. One-way ANOVA with Dunnett's multiple comparisons test compared to the LPS + vehicle group. *$P < 0.05$, **$P < 0.01$, ***$P < 0.001$, ****$P < 0.0001$. TCZ Tocilizumab.

© The Author(s)
